# Diagnostic performance of a circulating tumor DNA‐based blood test compared to fecal immunochemical test in colorectal cancer screening

**DOI:** 10.1002/cac2.70066

**Published:** 2025-09-30

**Authors:** Hermann Brenner, Teresa Seum, Megha Bhardwaj, Michael Hoffmeister

**Affiliations:** ^1^ Cancer Prevention Graduate School German Cancer Research Center (DKFZ) Heidelberg Germany; ^2^ Division of Clinical Epidemiology of Early Cancer Detection German Cancer Research Center (DKFZ) Heidelberg Germany; ^3^ Heidelberg Medical Faculty Heidelberg University Heidelberg Germany

**Abbreviations**
APCL: advanced precancerous lesion
cfDNA: cell‐free DNA
CRC: colorectal cancer
FIT: fecal immunochemical test
US: United States

1

Screening for colorectal cancer (CRC) has the potential to strongly reduce incidence and mortality of this common cancer [1, 2]. While fecal tests have long been established as non‐invasive CRC screening tests, an intensive search for blood‐based tests, which have been claimed to be easier to integrate into routine medical practice, is underway [3, 4]. Most recently, clinical validation of a circulating tumor DNA‐based blood test for the detection of patterns of CpG (cytosine followed by guanine) dinucleotide methylation has been reported from the PREEMPT CRC study, a large, United States (US) based screening study including 27,010 participants of screening colonoscopy [4]. The study was sponsored by the manufacturer and reported with limited details about the laboratory analyses. With a sensitivity of 79.2% (95% confidence interval [CI], 68.4%‐86.9%) for CRC detection and a specificity of 91.5% (95% CI, 91.2%‐91.9%) for advanced colorectal neoplasia, the test met the pre‐specified acceptance criteria, but the sensitivity for detecting advanced precancerous lesions (APCL) was low (12.5%). We aimed to compare the reported diagnostic performance of this blood‐based test with that of the fecal immunochemical test (FIT), the best established and globally most widely used non‐invasive CRC test [5], in a large population of screening colonoscopy participants from Germany.

Our analyses are based on data from the ongoing BLITZ study, whose design has been reported in detail elsewhere [6]. Briefly, participants undergoing screening colonoscopies were recruited in gastroenterology practices in Southern Germany and asked to complete a short questionnaire and provide a fecal sample prior to bowel preparation for the evaluation of non‐invasive CRC screening tests. Participants for this analysis were selected from 10,061 participants recruited between 2008 and 2020 for whom the same quantitative FIT (FOB Gold, Sentinel Diagnostics, Milano, Italy) was performed. The diagnostic performance characteristics of the FOB Gold assay were determined at the manufacturer‐recommended cutoff of 17.0 µg hemoglobin per gram of feces. To ensure comparability, we matched the inclusion and exclusion criteria as closely as possible to those of the PREEMPT CRC study, as outlined in the flow diagram in Supplementary Figure S1. We compared the sensitivity for detecting CRC (separately for any stage and stages I‐III), advanced precancerous lesions (APCL) and any advanced neoplasia (CRC or APCL), and the specificity of the blood test reported from the PREEMPT CRC study to the corresponding metrics for the FIT in the BLITZ study. APCL included in situ or high‐grade dysplasia, adenoma with villous growth pattern (≥ 25%), adenoma ≥ 1.0 cm, sessile serrated lesion ≥ 1.0 cm, and traditional serrated adenoma of any size. Differences were tested for statistical significance by two‐sided chi‐square tests at an alpha level of 0.05. In addition to analyses for the entire study population, we determined sensitivity and specificity for detecting any advanced neoplasia for subgroups defined by sex and age. Analyses were conducted using R version 4.4.0.

The main characteristics of the PREEMPT CRC and BLITZ study populations are presented in Supplementary Table S1. The proportion of men was higher in BLITZ (49.3%) than in PREEMPT CRC (44.2%, *P* < 0.001). Prevalences of CRC and APCL were also significantly higher (*P* < 0.001) in the BLITZ study (0.8% and 14.4% versus 0.3% and 9.5%), which included a higher proportion of older participants.

The sensitivity for APCL (23.7% versus 12.5%) and any advanced neoplasia (27.1% versus 14.3%), as well as the specificity (93.2% versus 91.5%), were all substantially higher for the FIT in BLITZ than for the blood test in PREEMPT CRC (all *P* < 0.001; Table 1). The sensitivity for CRC detection was also higher with the FIT than with the blood test (89.8% versus 79.2%), but this difference did not reach statistical significance. In subgroup‐specific analyses, sensitivity and specificity for detecting any advanced neoplasia were consistently higher for the FIT in BLITZ than for the blood test in PREEMPT CRC among men and women and among age groups 55‐64 and ≥ 65 years (*P* value for specificity among men = 0.027, all other *P* values < 0.001). Sensitivity and specificity were also slightly higher for FIT in the 45‐54 age group, but these differences were small and did not reach statistical significance. Within this age group, both FIT and the blood‐based test exhibited rather low sensitivity (13.5% versus 10.6%) but high specificity (94.3% versus 93.8%).

**TABLE 1:** Sensitivities and specificities of the circulating tumor DNA‐based blood test in the PREEMPT CRC study and of the FIT in the BLITZ study.

			PREEMPT CRC study^†^ (*n* = 27,010, 44.2% men, median age 57)		BLITZ study (*n* = 6,205, 49.3% men, median age 59)		
			Blood test		FIT^‡^	
Study population	Metric	Outcomes	No./total^§^	% (95% CI)	No./total^§^	% (95% CI)	*P*
Total	Positivity rate		2,443/27,010	9.0 (8.7‐9.4)	614/6,205	9.9 (9.2‐10.7)	0.037
	Sensitivity	CRC (stages I‐IV)	57/72	79.2 (68.4‐86.9)	44/49	89.8 (78.2‐95.6)	0.122
		CRC (stages I‐III)	45/60	75.0 (62.8‐84.2)	37/42	88.1 (75.0‐94.8)	0.101
		APCL	321/2,567	12.5 (11.3‐13.8)	211/891	23.7 (21.0‐26.6)	<0.001
		Any advanced neoplasia^¶^	378/2,639	14.3 (13.0‐15.7)	255/940	27.1 (24.4‐30.1)	<0.001
	Specificity	No advanced neoplasia^¶^	22,306/24,371	91.5 (91.2‐91.9)	4,906/5,265	93.2 (92.5‐93.8)	<0.001
Sex							
Men	Sensitivity	Any advanced neoplasia^¶^	210/1,450	14.5 (12.8‐16.4)	157/530	29.6 (25.9‐33.6)	<0.001
	Specificity	No advanced neoplasia^¶^	9,517/10,484	90.8 (90.2‐91.3)	2,331/2,529	92.2 (91.1‐93.2)	0.027
Women	Sensitivity	Any advanced neoplasia^¶^	168/1,189	14.1 (12.3‐16.2)	98/410	23.9 (20.0‐28.3)	<0.001
	Specificity	No advanced neoplasia^¶^	12,789/13,887	92.1 (91.6‐92.5)	2,575/2,736	94.1 (93.2‐94.9)	<0.001
Age, years							
45‐54	Sensitivity	Any advanced neoplasia^¶^	110/1,041	10.6 (8.9‐12.6)	10/74	13.5 (7.5‐23.1)	0.429
	Specificity	No advanced neoplasia^¶^	10,160/10,826	93.8 (93.4‐94.3)	396/420	94.3 (91.6‐96.1)	0.713
55‐64	Sensitivity	Any advanced neoplasia^¶^	132/909	14.5 (12.4‐17.0)	125/539	23.2 (19.8‐26.9)	<0.001
	Specificity	No advanced neoplasia^¶^	7,149/7,816	91.5 (90.8‐92.1)	3,096/3,312	93.5 (92.6‐94.3)	<0.001
≥ 65	Sensitivity	Any advanced neoplasia^¶^	136/689	19.7 (16.9‐22.9)	120/327	36.7 (31.7‐42.0)	<0.001
	Specificity	No advanced neoplasia^¶^	4,997/5,729	87.2 (86.3‐88.1)	1,414/1,533	92.2 (90.8‐93.5)	<0.001

Abbreviations: APCL, advanced preneoplastic lesion; CI, confidence interval; CRC, colorectal cancer; FIT, fecal immunochemical test; No., number.

^†^ Data extracted from Shaukat et al. [4]

^‡^ FIT positivity threshold recommended by the manufacturer (17.0 µg hemoglobin per gram of feces).

^§^ Proportions of overall positive test results (positivity rate), positive test results among those with the respective finding at colonoscopy (sensitivities), and negative test results among those with no finding of advanced neoplasia at colonoscopy (specificities).

^¶^ Advanced neoplasia comprised colorectal cancer and advanced precancerous lesions.

Despite the limitations of an indirect comparison (different study populations, different recruitment years, different prevalences), which we aimed to minimize by close matching of inclusion and exclusion criteria and outcome definitions, our comparative analysis of two large screening colonoscopy cohorts demonstrates that the investigational blood‐based test that was validated in the PREEMPT CRC study has worse sensitivity and specificity for detecting APCL than the FIT, the globally most widely used non‐invasive CRC screening test. Sensitivity for detecting CRC was similar for both tests. These results are in line with similar patterns we have previously demonstrated by comparing the diagnostic performance of the US Food and Drug Administration‐approved cell‐free DNA (cfDNA) blood‐based test from the ECLIPSE study, with that of FIT [3, 7].

In the youngest age group (45‐54 years), the diagnostic performance of the blood‐based test and FIT seemed to be comparable, suggesting that the blood‐based test may be a reasonable alternative to FIT for minimally invasive CRC screening in this age group. However, the number of study participants and advanced neoplasias was quite limited in this age group, particularly in the BLITZ study, which led to relatively wide confidence intervals for the sensitivity estimates. In another recent study from the US, sensitivity for detecting any advanced neoplasia among 40‐54‐year‐old participants was substantially higher for both FIT (35/144, 24.3%) and a next‐generation multitarget stool DNA test (49/144, 34.0%) [8], which suggests that the blood‐based test (sensitivity in age group 45‐54 in the PREEMPT CRC study: 10.6% [95% CI, 8.9%‐12.6%]) would also not be competitive to these stool tests at younger screening ages.

Another interesting observation, shared with findings from the cfDNA blood‐based test in the ECLIPSE study [3] and which should be followed up in further research, is the decline in specificity with increasing age of the blood DNA methylation‐based test in the PREEMPT CRC study. The authors of the PREEMPT CRC study hypothesize that this decline may be attributed to age‐specific alterations in DNA methylation signatures. An alternative hypothesis that should be followed up in further research might be that it may result from the presence of undetected cancer at a different site, in which case the blood‐based tests may have merits beyond CRC screening and positive test results might warrant follow‐up examination beyond colonoscopy.

In conclusion, our analysis confirmed that the sensitivity of the blood‐based test evaluated in the PREEMPT CRC study may be comparable to that of FIT for CRC, but not for APCL, and that specificity is worse. This blood‐based test, like a previously evaluated cell‐free DNA blood‐based test [3], should therefore not be considered as an equal alternative to or as a replacement for FIT‐based screening. The blood‐based tests may still be useful supplements to reach people whose aversion to stool‐based testing might otherwise deter them from CRC screening. Further research should aim at improving the sensitivity of blood‐based tests for detecting APCL.

## AUTHOR CONTRIBUTIONS

*Study design*: Hermann Brenner, Teresa Seum. *Statistical analyses*: Teresa Seum. *Manuscript writing*: Hermann Brenner. Critical revision of the manuscript for important intellectual content: all authors. All authors read and approved the final manuscript.

## CONFLICT OF INTEREST STATEMENT

The authors declare no conflicts of interest.

## FUNDING INFORMATION

This study was partly funded by grants from the German Research Council (DFG, grant No. BR1704/16‐1), the Federal Ministry of Education and Research (BMBF, grant No. 01KD2104A), and the German Cancer Aid (DKH, grants No. 70114735 and 70115864). The funding sources had no role in any aspect pertaining to the study.

## ETHICS APPROVAL AND CONSENT TO PARTICIPATE

The BLITZ study was approved by the ethics committees of the Heidelberg Medical Faculty of Heidelberg University (178/2005) and of the responsible state medical chambers. The study is registered in the German Clinical Trials Register (DRKS‐ID: DRKS00008737). Written informed consent was obtained from each participant.

## Supporting information



Supporting information

## Data Availability

The data that support the findings of this study may be made available upon reasonable request within the constraints set by informed consent and confidentiality regulations.
