# Supplementary material for: Diagnostic performance of a circulating tumor DNA‐based blood test compared to fecal immunochemical test in colorectal cancer screening
Source: Cancer Commun (Lond). 2025 Sep 30;45(11):1496–9. doi: 10.1002/cac2.70066 (PMC12629851; doi:10.1002/cac2.70066)
Supplement: Supplementary file 1 — Supporting information [file CAC2-45-1496-s001.docx]

**Supplementary Figure S1.** Flow diagram of participant selection from the BLITZ study. Abbreviations: CRC, colorectal cancer; FDR, first-degree relative; FIT, fecal immunochemical test; IBD, inflammatory bowel disease

**Supplementary Table S1**. Main characteristics of the study population in the PREEMPT CRC study and the BLITZ study.

| **Characteristics** | | **PREEMPT CRC study**^†^ | **BLITZ study** | ***P*-value** |
| --- | --- | --- | --- | --- |
| Total number of participants | | 27,010 | 6,205 | NA |
| Country, study period | | USA,  2021-2022 | Germany,  2008-2020 | NA |
| Sex | Men, *n* (%) | 11,934 (44.2) | 3,059 (49.3) | < 0.001 |
|  | Women, *n* (%) | 15,076 (55.8) | 3,146 (50.7) |  |
| Age, years | 45-49, *n* (%) | 2,968 (11.0) | 100 (1.6) | < 0.001 |
|  | 50-54, *n* (%) | 8,899 (32.9) | 394 (6.4) |  |
|  | 55-64, *n* (%) | 8,725 (32.3) | 3,851 (62.1) |  |
|  | 65-74, *n* (%) | 5,604 (20.7) | 1,591 (25.6) |  |
|  | ≥ 75, *n* (%) | 814 (3.0) | 269 (4.3) |  |
| Most advanced finding at colonoscopy | CRC, *n* (%) | 72 (0.3) | 49 (0.8) | < 0.001 |
|  | APCL, *n* (%)^‡^ | 2,567 (9.5) | 891 (14.4) |  |
|  | No advanced neoplasia, *n* (%)^§^ | 24,371 (90.2) | 5,265 (84.9) |  |

^†^Data extracted from Shaukat et al. [1]

^‡^Advanced precancerous lesions included carcinoma in situ or high-grade dysplasia, adenoma with villous growth pattern (≥ 25%), adenoma of 1.0 cm or greater, sessile serrated lesion of 1.0 cm or greater, and traditional serrated adenoma (any size)

^§^Advanced neoplasia comprised colorectal cancer and advanced precancerous lesions

Abbreviations: APCL, advanced preneoplastic lesion; CRC, colorectal cancer; NA, not applicable; SD, standard deviation

**Supplementary Reference**

1. Shaukat A, Burke CA, Chan AT, Grady WM, Gupta S, Katona BW, et al. Clinical Validation of a Circulating Tumor DNA-Based Blood Test to Screen for Colorectal Cancer. *JAMA.* 2025; **334**(1): 56-63.
